# Informal Science Education: Lifelong, Life-Wide, Life-Deep

**DOI:** 10.1371/journal.pbio.1001986

**Published:** 2014-11-04

**Authors:** Kalie Sacco, John H. Falk, James Bell

**Affiliations:** 1 Center for Advancement of Informal Science Education (CAISE), Association of Science-Technology Centers (ASTC), Washington, D.C., United States of America; 2 Oregon State University, Corvallis, Oregon, United States of America

## Abstract

Informal Science Education: Lifelong, Life-Wide, Life-Deep Informal science education cultivates diverse opportunities for lifelong learning outside of formal K-16 classroom settings, from museums to online media, often with the help of practicing scientists.

## Introduction

The words “science education” once evoked images of a professor in a white coat lecturing at a blackboard filled with equations or students in a lab conducting “experiments” with beakers and Bunsen burners. Such limited views of science education no longer hold today, when science, technology, engineering, and math (STEM) learning takes place in a wide variety of social dynamics and settings. Learning experiences might take the form of an observant walk on a family outing to a park, activities that impart lessons about biology at a summer camp or museum, or an evening at home watching *Cosmos* or other science television programs. These are just some of the experiences that constitute the expanding landscape of what is commonly called informal science education (ISE) in the United States.

There are many opportunities outside of formal K-16 classroom settings for doing and learning science. This is particularly important in the United States, where Americans learn most of their STEM knowledge or skills outside of school: 95% of the average American's lifetime is spent outside of the classroom, leaving ample time for informal science learning opportunities [1]. All learning, including STEM learning, is a continuous and cumulative process, primarily driven by individual needs and interests. The challenge is in connecting those needs and interests to this burgeoning learning landscape.

For the past seven years, the Center for Advancement of Informal Science Education (CAISE) has been building an infrastructure that the ISE field can use to document its projects, impacts, and research base. CAISE is a National Science Foundation (NSF)-funded center that provides resources and facilitates connections among researchers, practitioners, and other stakeholders who are currently involved in or hope to become involved in ISE activities and projects.

## What Is ISE?

To better understand the nature of ISE experiences, the National Research Council (NRC) *Learning Science in Informal Environments: People, Places, and Pursuits* [2] consensus volume provides distinct characterizations of learning “strands” based on findings from research and evaluation conducted in the field:

Strand 1: Experience excitement, interest, and motivation to learn about phenomena in the natural and physical world.

Strand 2: Come to generate, understand, remember, and use concepts, explanations, arguments, models, and facts related to science.

Strand 3: Manipulate, test, explore, predict, question, observe, and make sense of the natural and physical world.

Strand 4: Reflect on science as a way of knowing; on processes, concepts, and institutions of science; and on their own process of learning about phenomena.

Strand 5: Participate in scientific activities and learning practices with others, using scientific language and tools.

Strand 6: Think about themselves as science learners and develop an identity as someone who knows about, uses, and sometimes contributes to science.

There are a wide variety of ISE settings, or “sectors,” in which learners can experience one or all of these strands. Some of these settings include digital learning environments (websites, mobile apps, and online media); media (including TV, radio, and film); museums and science centers; libraries; zoos and aquariums; afterschool, summer, and extended learning settings such as hobby clubs; community organizations; universities; parks, gardens, nature centers, and arboretums; and public science events, cafes, and festivals.

One such project is the National Park Service's Trail of Time [3], which is an excellent example of how informal settings can support NRC's first three strands. Taking advantage of the existing paved Rim Trail on the most heavily visited section of the South Rim of Grand Canyon National Park, the Trail of Time is an interpretive walking timeline that focuses on Grand Canyon vistas and rocks and invites visitors to ponder, explore, and understand the magnitude of geologic time and the stories encoded in Grand Canyon's rock layers and landscapes. The timeline is marked by brass markers every meter, representing one million years of time, and viewing tubes and other interpretive materials help visitors connect the rocks visible in the Grand Canyon to their place along the geologic timeline.

First proposed in 1995 by University of New Mexico geology professor, Dr. Karl Karlstrom, the Trail was a collaborative effort between the National Park Service, the University of New Mexico, Arizona State University, and the University of Massachusetts, funded by the National Science Foundation. Completed in 2010, the Trail of Time is part of a research program in informal science education aimed at understanding and improving public understanding of the connection between human time scales and the “million-year heartbeat” of Earth.

Other informal learning experiences can range from the semiformal (like a child participating in a structured afterschool science camp) to completely “free choice” (such as a family visiting their city's science festival). Experiences can also be more contemplative (imagine casually perusing a museum exhibit) or participatory (like collecting data using a mobile app as a part of a citizen science program). Collectively and cumulatively, these experiences contribute to an individual's lifelong science learning.

The impacts of these settings have been documented in learning research and in the evaluation findings of individual projects; examples range from the incremental to the transformative. Many projects and their associated evaluation and research findings have been shared on the CAISE website, www.informalscience.org. These examples form a basis on which we can begin to understand how ISE can support lifelong learning.

## A Brief Survey of ISE Projects

ISE projects cover a range of approaches with different learning goals, each with their own challenges. Below, we describe just a fraction of ongoing projects, to illustrate the kinds of learning that can happen in informal learning settings and some of the challenges in developing experiences, as well as in measuring learning impacts in ISE.

### Citizen Science. *Hacienda La Esperanza Reserve*

Through ISE, participants can become engaged in the scientific enterprise, begin to see themselves as learners of and contributors to science, and use their own experiences to make sense of the natural world. Citizen science is a good example of this type of project. In citizen science projects, public audiences (that is, nonscience professionals) can collect data that contribute to a wider scientific research question or enterprise. One citizen science program developed by the Conservation Trust of Puerto Rico brings together local residents, visitors to Hacienda La Esperanza Reserve, and members of a community environmental project to explore local ecosystems and conservation issues facing these ecosystems [4].

Indicative of a *Learning Science in Informal Environments* Strand 6 outcome, participating local residents reported feeling a sense of ownership of the reserve and a recognition of the value of the scientific research that takes place there [5]. As is often the case in informal education experiences, participants self-select to participate, frequently because they already possess high levels of interest and often considerable prior knowledge. As a consequence, changes in these areas, Strands 1 and 2, respectively, are often modest. Assessing “learning” in these situations can be challenging using traditional assessment strategies because they are predicated on the assumption that learning is always based on “vertical” gains, e.g., from low knowledge to high knowledge, rather than the equally important and more typical “horizontal” gains, e.g., broadening and deepening of understanding [6]. Facilitators of learning experiences in informal contexts must be attentive to the fact that how people interact with scientific phenomena is likely to vary widely because of the heterogeneity of prior knowledge and experience.

### Media. *QUEST: Exploring Our Natural World*

Some practitioners have used ISE experiences to raise awareness and facilitate dialogue concerning regional science issues. For example, QUEST, a multimedia project [7] led by the KQED public media outlet in San Francisco, brought together a network of partners—including ISE organizations, science societies, and other public broadcasting networks—to produce online educational resources, television programs, radio reports, and professional development workshops to support dialogues about science content.

The “transmedia” breadth of QUEST allows the project to reach an age-diverse audience with a demonstrated interest in common, crosscutting themes: environmental issues, local concerns and organizations, high-quality content, and current science information. An independently conducted evaluation found that a major impact of QUEST's programming has been an increase in participants' engagement with science, allowing a wide range of individuals opportunities to explore, question, and make sense of the natural and physical world (Strand 3) [8]. The evaluation also identified a challenge that reflects the free-choice nature of ISE—that many participants cited a “lack of time” as a barrier to persistence in using KQED/QUEST online [9]. All designers of ISE experiences must grapple with competing demands placed on their audiences' time; nevertheless, informal opportunities can uniquely provide science content in the course of everyday lives—for example, listening to a science radio story over public broadcasting during a long commute.

### Salmon Camp Research Team

Because (in large part) learners choose to participate in ISE experiences, their motivation for doing so can be reinforced through positive experiences, leading to a feedback loop of increased interest in learning more about STEM. Hence, many ISE projects aim to catalyze or enhance motivation to engage in deeper learning and even pursue science academic and career trajectories (Strands 4, 5 and 6). For example, the Salmon Camp Research Team [10] targets Native American youth to explore local ecosystems, use technology to collect science data, and access traditional American Indian/Native Alaskan knowledge in addition to Western science framing.

One intended outcome of the project is to spark and sustain the interest of youth participants in STEM and Information Technology (IT) careers. Data from evaluation suggests that the program accomplishes that, particularly for high school students, whose exposure to college and university contacts through the program resulted in students pursuing higher education in the sciences [11].

## How Can Scientists Get Involved with ISE?

Scientists can leverage the strengths of evidence-based ISE practice to communicate their research to the public and engage with learners of all ages. For example, researchers might consider developing an informal learning activity or partner with an ISE program or project to help address the Broader Impacts merit criterion of their National Science Foundation awards.

In one such partnership, David Gruber, a marine biologist at Baruch College at the City University of New York, received NSF funding to investigate marine bioluminescence. Gruber and his collaborators dive more than 300 feet (100 meters) below the ocean's surface, using innovative high-resolution technology [12] to capture the deep-ocean organisms in action, despite low light levels. Diving in tropical locations such as Australia's Great Barrier Reef and the Cayman Islands, the team collects samples during their brief stay underwater and then analyzes their bioluminescent chemistry back in the laboratory.

In addition to publishing his research in some of the most prestigious journals, Gruber also helped to co-curate an exhibition at the American Museum of Natural History called *Creatures of Light: Nature's Bioluminescence*. In the exhibit, rocks, model jellyfish, and glow-in-the-dark toys illustrate fluorescence and phosphorescence. However, the bulk of the show focuses on creatures that produce light from internal chemical reactions. Unlike fluorescence, which requires an external source of shorter wavelength light, bioluminescence emits from within, and much of the exhibit content was based on discoveries Gruber made as part of his NSF research. This project demonstrates how researchers can enhance the dissemination of current science and their latest results by collaborating with an informal science institution that can transmit their work directly to the public.

Informal STEM education professionals can provide expertise and support for effective communication and engagement strategies and are often eager to connect their audiences with practicing scientists. Do you have a science center, natural history museum, or afterschool STEM learning program in your region? Consider reaching out to see if they would be interested in collaborating on an exhibit or program related to your current research topic. Other ways to get involved might include contributing a short piece on a local science issue for your regional public media organization or involving a community organization (from senior centers to Young Men's Christian Associations [YMCAs]) in data collection activities for your research. If you are based at a university, consider contacting undergraduate student organizations, on-campus childcare offices, or sponsored-project offices to find out about opportunities to connect with your local community.

Examples of these kinds of strategies and others can be found on the CAISE website: www.informalscience.org. CAISE recently developed an Outreach for Scientists page on the site to help scientists and directors of education/public outreach quickly find examples of ISE learning experiences sorted by scientific discipline: http://informalscience.org/about/informal-science-education/for-scientists. This resource provides an entry point to more than 1,600 project examples on InformalScience.org (many of which have associated evaluation reports). CAISE is continuously adding new examples to this free repository of resources, and we invite and welcome contributions from the research community.

## Abbreviations

CAISE: Center for Advancement of Informal Science Education
NSF: National Science Foundation
STEM: Science, Technology, Engineering, and Math
ISE: Informal Science Education
NRC: National Research Council
IT: Information Technology
YMCA: Young Men's Christian Association
